# “A Better Me for My Children”: A Call for Systemic Relational Family Work in Child and Youth Mental Health

**DOI:** 10.1177/10443894241261743

**Published:** 2024-09-03

**Authors:** Jane Elizabeth Sanders, Ariel Seale, Lauren Wilton, Emily Carrothers, Mackenzie Siddall, Zenaida Pereira, Elora Watson, Kendra Bernardi, M. K. Arundel, Rick Csiernik

**Affiliations:** 1 BA, BSW, MSW, PhD, RSW, associate professor, King’s University College at Western University Canada, London, ON, Canada; 2 BSW, RSW, research coordinator, King’s University College at Western University Canada, London, ON, Canada; 3 BSW, MSW, RSW, research assistant, King’s University College at Western University Canada, London, ON, Canada; 4 MSW, RSW, SAFE clinical supervisor, King’s University College at Western University Canada, London, ON, Canada; 5 BSW, MSW, RSW, research assistant, King’s University College at Western University Canada, London, ON, Canada; 6 MSW, Director of SAFE and KCSC and manager of professional practicum education, King’s University College at Western University Canada, London, ON, Canada; 7 BSc, BSW, MSW, PhD, RSW, professor, School of Social Work, King’s University College at Western University Canada, London, ON, Canada

**Keywords:** systemic, relational, family therapy, children’s mental health, social work education

## Abstract

Despite an established body of knowledge supporting the efficacy of systemic and relational approaches in children’s mental health care, there are mounting barriers to family involvement. The current reflexive thematic analysis included data from parents, social work students, university faculty and field professionals, and school board professionals (*n* = 65) to understand the mental health needs of parents and families. The findings exemplify the importance of systemic and relational family work for parents supporting their child and family’s mental health. We found a pre-existing and deepening gap in the accessibility of such supports since COVID-19. Implications include a call to renew systems thinking in children’s mental health, a systemic re-shift toward family service organizations including options for multiple members of a family, and education in social work that fosters systemic and relational family approaches to children’s mental health.

## Introduction

An established body of knowledge supports the efficacy of a systemic approach in children’s mental health care (Carr, 2019; Haine-Schlagel & Walsh, 2015; Profe & Wild, 2017). A systemic approach contextualizes an individual within their environment. It recognizes the importance of the multiple layers of influence, including those most proximal (microsystem), such as family, for support and intervention (Ahmed et al., 2018; Bronfenbrenner, 1977). And yet, despite the well-known benefits, family involvement in children’s mental health has given way to a focus on individually delivered services, and specialized and manualized forms of therapy (Lebow, 2014). Organizational cultures can limit family involvement and reflect reservations related to power relations, fear of negative outcomes, and a perspective that prioritizes an exclusive patient–professional relationship (Eassom et al., 2014). Family service agencies now mostly practice with individuals, one-way mirrors are rarely available, and supervision has been reduced to administrative requirements (Imber-Black, 2014).

The objective of the current qualitative study was to understand the mental health needs of parents and families. Data were collected from parents, school board professionals, social work students, and professionals from our School of Social Work (*n* = 65). The value of parent-focused services was clear, for parents themselves, their children, and their families. Equally clear was the profound and growing gap in the availability of such services.

### Evidence-Informed Children’s Mental Health Involves Families

Individuals in families affect one another, and family processes influence individuals just as individuals affect family processes (Lebow, 2014). Factors of family functioning, such as family rules, promoting emotional expression, and involvement among family members are related to adolescent symptomology, emphasizing the importance of family involvement in psychosocial programs for children and youth (Caño González & Rodríguez-Naranjo, 2024). Family involvement in treatment has been established as beneficial across a range of concerns, including addiction (Esteban et al., 2023; Kourgiantakis et al., 2021), eating disorders (Jewell et al., 2016; Loeb & Dimitropoulos, 2023), support for Queer youth (Harvey & Stone Fish, 2015; Malpas, 2011), family violence (Vincent, 2018), child welfare (Augsberger et al., 2023), and improving academic outcomes (Wang et al., 2014). Systemic interventions involving families effectively address a range of concerns, including sleep, feeding, and attachment problems in infancy; recovery from child maltreatment; conduct problems, emotional concerns, eating disorders, somatic difficulties, and first episode psychosis (Carr, 2019). A meta-analytic review highlighted that including parents in the psychotherapeutic treatment of children had advantages beyond outcomes achieved by individual child therapies (Dowell & Ogles, 2010). Furthermore, a review by Hogue and colleagues (2014) identified the benefits of specific family models beyond those of popular and relatively well-researched approaches, such as cognitive behavioral therapy (Hofmann et al., 2012). Not only is there a positive impact on outcomes when parents are involved in child and adolescent mental health interventions (Waid & Kelly, 2020) but meaningful parent engagement and empowerment in mental health services also supports parents (Bode et al., 2016).

### Barriers to Family Mental Health

Barriers to child and adolescent mental health care exist at the client, service provider, community, and health system level (Waid & Kelly, 2020). In Ontario, the location of this study, 18%–22% of children and youth met the criteria for a mental health disorder but under one third had contact with a mental health provider (Georgiades et al., 2019). However, 4 in 10 parents report not receiving adequate timely help for their children due to long waitlists and services that do not meet the unique needs of their family (Children’s Mental Health Ontario, 2020; Georgiades et al., 2019). When there are such profound gaps in service provision, families cover the deficiencies, which in turn leads to overload and reduced quality of life for caregivers (Carbonell et al., 2020). The burden of care that falls on families, results in a quarter reporting missing work to manage the increased complexity of parenting and childcare responsibilities (Children’s Mental Health Ontario, 2020). In turn, those who are parenting children with mental health concerns are more likely to present with their own mental health issues, furthering the detrimental impact on the family and decreasing outcomes for youth, yet few parents and caregivers receive support for themselves (Acri & Hoagwood, 2015).

To add complexity, at the time of writing, families were still reeling from the profound global impact of the COVID-19 pandemic which saw increased isolation and restriction of services spanning years (Imran et al., 2020; Palacio-Ortiz et al., 2020; Spinelli et al., 2020; Waite et al., 2021). During COVID-19, youth mental health concerns increased and support decreased, leaving families to take on the responsibility of caring for youth with little support or recognition (Kourgiantakis et al., 2022).

Furthermore, responsibility for child and family mental health disproportionately falls on mothers and female-identified caregivers. This was exacerbated during the pandemic when women were often subjected to a triple workday consisting of their regular employment, engaging in domestic labor, with the addition of child-rearing and supplementing education while learning at home (Jasrotia & Meena, 2021; Peck, 2021; Sharma & Vaish, 2020). There is an association between increased household demands for women with increasing physical, emotional, and psychological stress (Jasrotia & Meena, 2021; Sharma & Vaish, 2020). The identified lack of support for parents disproportionately affects mothers and therefore is a significant equity issue. Moreover, these barriers to services are even more profound for marginalized, racialized, newcomer, and female-headed single-parent families (Chen et al., 2012; Watson et al., 2020).

Such barriers cause tension and stress in caregivers’ attempts to access services, advocate, and provide mental health support for their children (Boydell et al., 2006; Miller et al., 2017). This is a systemic issue, as parenting responsibilities are compounded by system navigation struggles, stigma and blame, a lack of social support, and social isolation (Acri & Hoagwood, 2015). In a cyclical process, high parental stress acts as a barrier to a parent’s active and meaningful engagement in their child’s treatment (Bode et al., 2016). Children of parents with mental health concerns are twice as likely to develop mental illness themselves (Wahl et al., 2017; Wolicki et al., 2021). Therefore, attending to parental mental health is essential within the context of their child’s treatment.

### Context of the Study

At the time of widespread social limitations brought on for public safety during the COVID-19 pandemic, our School of Social Work at King’s University College at Western Canada (King’s) partnered with the local public school board, Thames Valley District School Board, to create a virtual agency with service delivered by bachelor and master social work students in practicum and supervised by a registered social worker. The Support and Aid to Families Electronically (SAFE) program was developed to deliver virtual services for parents and caregivers of school-aged children through no-cost, no-waitlist, and low-barrier support (Sanders et al., 2023, 2024). SAFE is a university–community partnership designed in line with an integrated knowledge translation (IKT) approach to generate evidence-based, relevant, and usable knowledge, in which knowledge users identified a problematic gap in support and continued to be involved as members of the research and writing team along with the SAFE students (S. Bowen & Graham, 2009; Preyde et al., 2013). The number of families receiving services through SAFE increased from 35 in the first year to 109 in the second year, illustrating the profound gap in accessible family support.

### Theoretical Foundation

The current study is informed by a systemic understanding of the family and the importance of the environment in which families reside. Ecological theory considers an individual in multiple contexts, from the direct environment in which they grow, expanding outward to include larger social and structural settings (Bronfenbrenner, 1977). Family system theory emphasizes that a family is a complex unit that is intensely emotionally connected and this dynamic has a profound effect on each member; therefore, an individual is best understood in relationship within the family (M. Bowen, 1978; Haefner, 2014; Simon et al., 2019).

Family therapy is defined as “any psychotherapeutic endeavor that explicitly focuses on altering the interactions between or among family members and seeks to improve the functioning of the family as a unit, or its subsystems, and/or the functioning of individual members of the family” (Gurman et al., 1986, p. 565). Systemic practice includes family therapy and other family-based interventions, such as parent training, parent-implemented behavior programs, multisystemic therapy, and treatment foster care, those which engage family members and wider networks in the process of problem resolution for a member of the family (Carr, 2019).

Our students were encouraged to align their work with the practice model that fit the needs of the client and fostered the therapeutic relationship. Therefore, in line with graduate programs’ requirement to teach a full range of models (Imber-Black, 2014), our approach to the work was not aligned with any one model or approach, rather students engaged with parents to understand their needs and develop a formulation-based, client-centered, and evidence-informed intervention plan (Drisko & Grady, 2015). Although our students were not explicitly developing whole family therapy skills, the approach across our School of Social Work is systemic and the service delivery in SAFE generally fell within the definition of relational family therapy promoted in the work by Breunlin and Jacobsen (2014), in which a practitioner works with a subset of the family or an individual but with a systemic lens. Moreover, our program is embedded in a critical intersectional awareness of social justice (Collins, 2019; Crenshaw, 1991) and was heavily informed by the three movements identified in the work by de Paula-Ravagnani and colleagues (2023) as influencing family therapy: postmodernity, social constructionism, and research on common factors (see also Dickerson, 2014).

### The Current Study

The objective of the current qualitative study, using reflexive thematic analysis (TA) (Braun & Clarke, 2021), was to understand the mental health needs of parents and families who participated in the first 2 years of the SAFE program. The following research questions guided the inductive analysis: (a) What mental health support do families need? (b) How effective did families feel support was at the time of this study? The current reflexive TA (Braun & Clarke, 2021) is part of a larger study of the SAFE program conducted in 2021–2022 in Ontario. Other analyses have focused on the feasibility of the pilot study for SAFE (Sanders et al., 2024), while this qualitative analysis of the data is focused on the experiences of parents and families. This overarching research used participatory action research (PAR) and the above noted integration of an IKT approach. PAR supports community member involvement in the research, in this case, social work students as service providers, to develop research that is most relevant and appropriate (Csiernik et al., 2018; Dickson et al., 2020; Sperstad et al., 2020).

## Method

Reflexive TA of semi-structured qualitative interviews, focus groups, and qualitative surveys was used to allow meaningful opportunities for study participants to express their experiences (Braun & Clarke, 2021). Reflexive TA is an appropriate method to organize, analyze, and conceptualize patterns found in qualitative data. (Braun & Clarke, 2006, 2021). Reflexive TA is a postmodern constructivist approach that views the subjectivity of the researchers as inevitable and an asset (Braun & Clarke, 2021). The themes detailed in our results section were reflexively and inductively (rather than deductively) derived from the data (Braun & Clarke, 2021).

### Participants

There were 65 participants involved in the study between year 1 (Y1) January 2021–June 2021 and year 2 (Y2) September 2021–August 2022. This included four participant subgroups to increase rigor through triangulation of data sources. However, 4 parents who were service users of SAFE in Y1, and 13 in Y2 (*n* = 17) participated; 8 SAFE social work students who delivered the service to families were involved in Y1 and 4 in Y2 (*n* = 12); 4 professionals from our School of Social Work at King’s who were involved in developing and running the SAFE program in Y1, and 2 additional in Y2 (*n* = 6); and 22 referring district school board professionals including social workers, counselors, and psychologists in Y1, and 8 in Y2 (*n* = 30) participated in the study. The vast majority of participants across all subgroups identified as female (except two male social work students and two male school board professionals) and white (except one student who identified as Black, one as white and Indigenous, and one as Black, Greek, French, and Scottish).

#### Participant Recruitment

Participants were a convenience sample recruited from the four subgroups. This included all parents (P) who were involved in the SAFE program from January 2021 to August 2022; all social work students (ST) placed in the SAFE practicum during that time frame, all professionals from our School of Social Work who were involved in the SAFE program (K), and all local school board professionals who were eligible to make a referral to SAFE (SB) at that time. Recruitment was completed at the point of information power, or the saturation of codes (Braun & Clarke, 2021). Informed consent letters were distributed to parents by the intake workers. Informed consent forms and a link to a Qualtrics survey were emailed to the students, King’s professionals, and school board professionals. Parent participants each received a US$25 gift card for their participation. Research ethics approval was granted through the supporting university.

#### Researcher Description

The lead author, a cisgendered middle-aged white female, was a child and family therapist, school social worker, and clinical supervisor for more than 25 years and almost 10 years conducting research in these areas. The focus of this manuscript constructively developed out of concerns observed through this long career. Consistent with IKT and PAR, the research team consisted of research assistants, students placed in SAFE, the Manager of Professional Practicum Education, a field supervisor, and professors in the School of Social Work.

### Data Collection Procedures

Data were primarily collected through individual, semi-structured, in-depth interviews (I; *n* = 31) and focus groups (FG; *n* = 3) each lasting 60–90 min. Given the ongoing stress related to pandemic restrictions at the time, Qualtrics surveys with open-ended text questions (S; *n* = 14) were included for those unable to attend an interview or focus group yet interested in participating. This was particularly important for the district school board professionals who were stretched beyond capacity at this time (Sanders et al., 2024). Interviews and focus groups were conducted over Zoom and audio-recorded with permission. These were transcribed and analyzed along with the qualitative content from the online surveys. Semi-structured questions were adjusted for each of the participant groups but focused on three main areas: the impact of COVID-19, the efficacy of SAFE services, and accessibility of community services.

### Data Analysis

As per the first step of TA (Braun & Clarke, 2006, 2021), four research team members familiarized themselves with the data by reading and re-reading each whole interview to become immersed in its content. During Step 2, data were coded by two independent coders, with a focus on inductive coding categories. The use of multiple coders facilitated engaged discussion of the data and development of themes, an important element of effective reflexive TA (Braun & Clarke, 2021) and allowed meaningful engagement of all members of the research team in line with our overarching IKT and PAR approach. As per our methodology, we did not strive for interrater reliability or consensus (Braun & Clarke, 2021). Each code label represented singular ideas derived from sections of the data that were of analytic interest. In step three, codes and potential themes were identified for each participant subset. Although the data from each subgroup were coded, for this analysis, the data and themes derived from the analysis of the parent interviews were prioritized and the data from the remaining three subgroups were used similarly to constant comparison in constructivist grounded theory (Charmaz, 2014) and as a form of triangulation. The importance of support to families and the growing gap in this support were clear from the outset. In the fourth and fifth steps, all themes were reviewed, inconsistencies were discussed, and the team collaboratively defined and refined the themes and co-occurring subthemes of the entire dataset. As per Step 6, the analysis was finalized by the lead researcher whose subjectivity was an asset used to structure the analysis in the current manuscript (Braun & Clarke, 2021). To reduce coder bias and further enhance reliability, trustworthiness, and credibility, an audit trail was kept to document research decisions (Anastas, 2004; Nowell et al., 2017).

## Results

The findings of the current study highlight the importance of supporting parents and families as a whole system, even when the mental health of children and youth is the priority; as one parent articulated, “therapy made me a better me for my children” (Y1-I-P01). However, despite the long-standing evidence base for systemic and family approaches in children’s mental health, the current findings identify an increasing gap in support for parents and families that has been exacerbated since the COVID-19 pandemic.

### Relational Support to Parents

A major theme that emerged was the value of providing support to parents for the health of the family system, including the children and youth in that system. All referrals to the SAFE program were made through the school board and therefore were connected to an identified child or youth. However, for many parents, it was the direct support they received that led to the most change, “It wasn’t somebody stepping in and doing services for him or testing for him. It was a support piece for me to help him. And I think that’s the big difference” (Y1-I-P06). University professionals agreed, as one noted “I cannot stress enough that you cannot affect the kind of change that we want to be able to see happen for these families and these children by only working with the children” (Y1-I-K02).

The family work was often complex and relational, as participants described, “I was able to talk about my relationship with my husband and my husband’s relationship with my son and how that might be impacting things and how I can help mediate their relationship” (Y1-I-P06). In some situations, parents identified that they needed help to, “learn skills to help their children through different struggles that impact various aspects of their growth and wellbeing as well as the family” (Y2-S-P03). One parent described the process as “just allowed me to allow the emotion that I was feeling. . .and then make a rational decision. . .am I making the, what did he call it, catastrophic thinking?. . .Or is it just emotional response right now and that’s causing this thinking” (Y2-I-P23).

#### Parent Mental Health is Child Mental Health

Parent and child mental health was seen as reciprocally connected, with one student noting,

When you’re the parent, you’re trying to hold it all together and make the best decisions you can, but when you’re fearful and ridden with anxiety, that’s going to impact the entire family system. Our children are always watching, they see these things, they notice our mood changes, they notice when we’re more worried about something. (Y2-I-ST10)

Another parent observed that in their family system, the most effective change started with themselves, “as parents, we can’t change the teenager, so how can we change the way we’re thinking about the situation or responding” (Y2-I-P16). As an example, a King’s professional observed:

I think we had five or six gender questioning kids, gender curious kids whose parents came to us for supports and whose parents left really feeling like they had a more fulsome understanding of who their child was and what their child was asking in terms of pronouns. (Y2-I-K05)

#### Supporting Parents Reduced Stress in the Family

With the complex issues families faced, the emotional burden fell on parents, particularly mothers, and support for them reduced stress in the family system, as one parent observed, “I found that my mental health had the greatest impact on the mental health and emotional regulation of the entire household” (Y2-S-P03). Parents recognized how their children were affected by their stress:

[they] could see that we were stressed and they held back on even when I asked them things and actively approached them, they didn’t tell me how deeply things were affecting them, in the hopes that they wouldn’t stress us out more. But I think that the house of cards of everything falling down at the end was a trigger and it just all rushed out. (Y2-I-P12)

As parents articulated “[we] have to be able to regulate ourselves if we’re going to help our teenagers regulate, right” (Y2-I-P16) and they noted, “sometimes it’s so hard to get out of your own head or your own heart, in a lot of these issues” (Y2-I-P23). Moreover, parents ultimately felt responsible “I would argue parents are just drowning in responsibility and worry for the now and the future” (Y2-I-P16). We were struck by the importance of dedicated support when going through significant family turmoil and that this was a new experience for parents that created change within a family, “the fact is. . .even to your closest friend, you can only talk so much about your child punching you in the face or being lured by sex traffickers. No one wants to hear this. This is uncomfortable” (Y2-I-P16).

Ultimately, the parents in this study had developed an understanding of the link between their mental health and their ability to support their children, in all the ways that parenting requires, “it was a huge thing to me to have someone come into my life and say, it’s okay to feel like you need to take care of yourself, it’s okay to admit that you need help. . .I realize that I was doing more harm than good not caring for myself” (Y2-I-P12), and as one parent was told by their SAFE student counselor, “If you’re okay, then he’s okay. You have to be okay for him to be okay” (Y2-I-P23). Interestingly, parents felt this was something they wanted to model to their children, again highlighting the connection between parent and child mental health.

### Limited Mental Health Support for Children and Families

A second theme was the limited access to services for children and families. We were struck by the consistency of parents’ stories of struggling to access services for their children, “it took me a year to get him set up with the (school) counsellor” (Y1-I-P01). Barriers to adequate service included “long waitlists and challenges even getting approved or getting a hold of someone who would put them on the waitlist” (Y1-FG-ST01), and caseloads that service providers “can’t necessarily manage” (Y1-FG-ST02). In addition, parents were increasingly concerned about the cost of services, particularly if their children required specialized testing or assessment, as one parent identified, “learning disability testing, the dyslexia testing. You can do that all privately, but it costs a fortune” (Y1-I-P06).

#### Parents Prioritizing Support for Their Child Over Themselves

If child services were inaccessible, services to parents were even more so, as illustrated by this parent’s experience,

and the person literally, not laughed in a mean way, but did a horrified chuckle about how I was probably more likely to find help for my kid long before I found any sort of help for myself. So, at that time I was just like, oh, well, then I guess I won’t be seeking out anything for myself. . . (Y2-I-P12)

School board professionals acknowledged this, with one stating “a lot of our parents are told that they need to work on things, but then not given places to access that” (Y2-I-SB03). Families are interconnected systems, and yet our support systems appear less and less structured to support children and parents as a unit, for example, “I had been to [services for abused women] at the very first, but that didn’t work out very well because I was in charge of five kids and I was alone” (Y2-I-P12). As a result, parents prioritized their children’s mental health needs, “parents are okay with that because they have shifted and said ‘I want to focus on their mental health and their well-being, and that’s what I can manage right now’” (Y2-I-SB02). This is not a decision that people should be forced to make, as one parent articulated:

we push our trauma down and we go to work. We push our trauma down and we soldier on and all these kind of things. But even as adults, we know that that isn’t right. But for kids, somebody has to help these kids. (Y2-I-P12)

It is important to note that all parent participants in this study identified as women, most of the referrals to SAFE were mothers and most of the service providers were women (Sanders et al., 2024), and when parent participants spoke of the need to push trauma down they were referring to gender-based trauma, such as intimate partner violence and sex-trafficking. As one student explained:

having children at home who are also struggling with their mental health, and now you’re struggling with your mental health, it seems like a lot of parents were really feeling like they’re not able to provide the support that they needed or that they wanted to for their children, and also to maintain their own wellness at the same time. (Y2-I-ST10)

#### Growing Gaps in Service Accessibility

The concerns identified in our study highlighted long-standing and growing gaps, as one King’s professional pointed out, “when you’re looking at the data of mental health issues pre-COVID-19, addiction issues, discord and conflict in the home, parenting strategies, they were already in existence. And social work in the schools was already overworked with issues” then, with COVID-19, “the one free mental health service for youth and families really pulled back” (Y1-I-K04). As one school board professional noted “as we got into the second and the third. . .waves, I think I saw the impact and the need increase” (SB-Y2-I-02). Waitlists are a particularly important measure of accessibility, as pointed out by several participants, “they’re waiting almost two years sometimes for counselling. Which is not okay” (Y2-I-SB04).

In the second-year data, mental health concerns and daily stressors appear to have increased, as many participants observed,

return to work and the increased workloads, coupled with housing prices and inflation. . .had a lot of parents really struggling with a lot of mental health, not sleeping very well, not eating very well, not engaging with social supports even if they could. (Y2-I-K05)

In addition, school board professionals observed, “increases in hopelessness, and people not feeling like this is going to get better” (Y2-I-SB02). Indeed, as one parent noted, “it’s pretty difficult on the whole to find mental health support, right now, that’s consistent or actually has the ability to get you where you need” (Y2-I-P15). This second theme of limited resources further highlights our first theme of the value of services for families. The participants in this study stressed that such support led to real change amid increasing stress.

## Discussion

The focus of this study is on furthering the evidence base and supporting a clinical focus on effective practice with multiple members of a family system, particularly parents, guardians, and caregivers, even when the overall focus is on the child. The value of parent-focused services was clear. Equally clear was the profound gap in the availability of such services. Through inductively generated reflexive TA, we found important benefits for families when services are offered to parents. Our first overarching theme highlights the value of relational support to parents. This theme was furthered by the subthemes, parent mental health is child mental health, and supporting parents reduces family stress. Our second theme names the impact of limited access to mental health support in general and more acutely to parents. Subthemes highlight that amid limited resources, parents, primarily mothers, will prioritize support for their child over themselves and that there are growing gaps in service availability as we move past the acute phase of COVID-19.

Knowing the impact of larger system issues on families (Lebow, 2020), family support, parental involvement, and collective family, mental health has never been as important (Kourgiantakis et al., 2022). Moreover, the continually evolving repercussions of COVID-19 (Schmidt et al., 2021) including the profound increase in need partnered with a decrease in service provision (Kourgiantakis et al., 2022) serve as a stark illustration of how ill-prepared we are for future such events that are equally likely to be shaped by racism, classism, and the climate crisis (Watson et al., 2020).

The benefits of family approaches to child mental health are rearticulated in the current study through the voices of those who participated. Despite the strength of the literature identifying the importance of family involvement in child and youth mental health, our findings illustrate how shrinking resources and narrowly focused and individualized supports create barriers to systemic approaches and parental involvement, forcing parents to prioritize their children’s mental health over their own and eschew supporting the family as a unit. Not least, barriers to service include a pre-existing and profound lack of services (Georgiades et al., 2019), organizational culture, and policy that limits family involvement (Eassom et al., 2014) compounded by increasing service complexities and challenges of maintaining service during the social isolation of the pandemic (Ashcroft et al., 2022).

Our study is consistent with extant literature identifying that families cover the deficiencies in service provision, overloading caregivers and affecting quality of life (Carbonell et al., 2020). Moreover, the responsibility for family mental health disproportionately falls on mothers and those who identify as female (Goldin, 2022; Jasrotia & Meena, 2021; Peck, 2021; Sharma & Vaish, 2020). The stress identified by mothers in our study and the forced choice of sacrificing their own support needs exemplify how the lack of support for parents is a significant equity issue.

### Implications

The implications from our study are threefold. First, we hope to see a renewed focus on systems thinking that underscores the benefit of parental support to the health of an entire family and community. The value of working systemically with the awareness of multiple members appears to have been overshadowed by specific and manualized interventions that are often applied to individuals (Lebow, 2014). As noted, this is fostered by narrow conceptions of evidence-based practice and organizational cultures that limit family involvement (Eassom et al., 2014; Imber-Black, 2014).

This in turn leads to our second implication which is the need for a systemic re-shift within organizations toward options for multiple members of a family and direct support to parents. Our findings encourage organizational change in culture and policy that fosters a collaborative and client-centered orientation, including relational and systemic approaches as viable options available to children and families as a shared goal across all members of a clinical team, including agency leaders (Eassom et al., 2014). To achieve this would require an examination of the current systems supporting family mental health, including in schools, community mental health, and child welfare, and a deep examination of the resource needs across the system. For example, barriers to the implementation of whole family support in programs include adequate training for staff and resources (Selick et al., 2017). We concur with Acri and Hoagwood’s (2015) recommendations that service providers, administrative structures, and reimbursement strategies be strengthened and connected to the service needs of caregivers and children to enhance family outcomes.

Third, clinicians with training in family work are more likely to work with families, feel more competent in this work, and have more positive attitudes toward family involvement (Kim & Salyers, 2008). The training of family therapists and clinicians who do not explicitly identify as family therapists, but who are interested in child and family mental health, is primarily located in degree-granting programs such as ours. Therefore, systemically attuned and relationally based family mental health services require that degree-granting programs provide educational opportunities with our first two implications in mind. As Breunlin and Jacobsen (2014) identified, the relational approach to family therapy is most likely to be practiced in conjunction with individual therapies that are currently popular. As co-authors who are educators, students, and practitioners, we hope to help orient students to the import of systems thinking and the value of family involvement whether explicitly working systemically or just interested in what works (Imber-Black, 2014). This is foundational to the continuation of systemic thinking detailed in our first implication and is necessary to ensure emerging practitioners are attuned to the value and skill of systemic and family-oriented practice to maintain these as viable options within family-serving organizations.

The findings of this study have important implications for all direct practice programs to foster a systemic and relational understanding of child and family mental health. We view this as foundational for fostering awareness and understanding of the importance of family processes and future family therapy skill development. Furthermore, we view it as essential that such training occurs within a critical and systemic understanding of contemporary efforts to decolonize and diversify family approaches (D’Arrigo-Patrick et al., 2017; Watson et al., 2020).

### Strengths and Limitations

As with all qualitative research, these results are not intended to be generalizable beyond the study group, but it is worth reiterating that the findings are derived from data collected from four groups involved in various ways with one social work support program. Moreover, the timing of our findings must be taken into consideration in interpreting the results. The need to focus on parent mental health, however, is not unique to COVID-19 and we believe the implications highlighted in this study will be relevant well beyond COVID-19 and provide learning for future crises.

### Recommendations for Future Research

The findings of the current study give direction for future emphasis on the importance of accessible, systemic, relational, and family-focused services for children and families. While the efficacy and importance of family involvement and systemic approach to child, youth, and family mental health have long been understood research and in turn practice focus in this area has waned. We recommend a renewal of study on the importance of a systemic approach to child services including school-based services, child welfare, youth justice, and child and youth mental health. Specifically, we would recommend a synthesis of analysis across qualitative studies to capture the experiences of families in other contexts. This will help build our knowledge of the global impact of the identified gap in parental involvement in child and youth services.

## Conclusion

The current study highlights the importance of working systemically to include and support families in mental health services to children and youth. Although the benefits of a family approach have been long understood, the findings of this study and the existing literature highlight that shrinking resources and organizational culture can create barriers to parental involvement. We hope to add the voices of our participants to the call for a systemic reorientation toward family involvement that is further supported through social work degree-granting programs such as ours.
